# Is generative artificial intelligence literacy important? A study on the factors influencing teacher’s professional flourishing

**DOI:** 10.3389/fpsyg.2026.1768712

**Published:** 2026-03-26

**Authors:** Linfang Guo

**Affiliations:** 1 Faculty of Education, Henan Normal University, Xinxiang, China; 2 Henan Police College, Zhengzhou, China

**Keywords:** generative artificial intelligence literacy, organizational emotional support, research output capacity, teacher professional development, teacher’s professional flourishing

## Abstract

This study, based on the theoretical frameworks of self-determination theory and social support theory, uses a stratified random sampling method and a questionnaire survey to collect data from 499 university teachers at a public university in Jinan. It innovatively explores the impact of personal ability factors, such as generative artificial intelligence literacy, and external support factors on teachers’ sense of professional flourishing. Through mediation analysis, the study reveals that teachers’ professional flourishing is influenced not only by external support factors such as the research atmosphere, teaching atmosphere, and organizational emotional support but also by internal factors, including teaching efficiency and generative artificial intelligence literacy. Furthermore, the study identifies four mediating pathways. Generative artificial intelligence literacy significantly improves teachers’ professional flourishing by enhancing teaching efficiency, while the research atmosphere, teaching atmosphere, and organizational emotional support also indirectly promote teachers’ professional flourishing through increased teaching efficiency. This study provides a new perspective for educational practice, revealing the importance of the collaborative effect of both internal and external factors, such as generative artificial intelligence literacy, on teachers’ professional flourishing.

## 1 Introduction

Over the past few decades, as Chinese universities have introduced new performance management systems such as “non-promotion leads to dismissal,” “bottom-ranking elimination,” “target-based evaluation,” and “merit-based appointments,” the internal performance governance mechanisms in these institutions have gradually been reshaped. The ideologies of New Managerialism and New Public Management, which share a common origin, have provided the legitimacy for the responsibility and pursuit of performance. All these new performance management systems are aimed at individual achievement and high performance output. Driven by this performance-oriented approach, the excessive pursuit of outstanding results has become the norm among stakeholders in universities. It is even regarded as an unspoken rule for enhancing the overall strength of universities and competing for a new round of “Double First Class” status (Xu, 2025). However, in this environment of high-performance pressure, many teachers face significant pressure that may undermine their job satisfaction and overall wellbeing, thereby affecting their sense of professional flourishing.

Professional flourishing refers to the sense of achievement, satisfaction, and mental wellbeing that teachers experience throughout their careers (OECD, 2024). It is a core dimension of teachers’ mental health and professional development. Professional flourishing among teachers is not only a key dimension of their mental health and career development but also a solid foundation for job satisfaction, teaching effectiveness, and student learning outcomes. Under the implementation of new performance management systems in Chinese universities, teachers are facing increasingly intense competitive pressure. This pressure comes not only from external performance evaluation systems but also from internal self-expectations and career goals. However, how to maintain a positive sense of professional flourishing in such a high-pressure environment and how to find inner achievement and satisfaction in the pursuit of results have become difficult challenges for many educators. Moreover, with the rapid development of generative artificial intelligence (AI) technology, teachers’ sense of professional flourishing faces new challenges and opportunities. An increasing number of teachers are beginning to worry whether they will be replaced by generative AI and whether they will still be able to play a key role in teaching. This anxiety has not only intensified teachers’ professional pressure but also, to some extent, affected their sense of professional flourishing. Generative artificial intelligence has introduced new educational tools and resources, but how to utilize this emerging technology in an environment filled with uncertainty and anxiety has become an important issue in teachers’ professional development.

Generative artificial intelligence literacy is not only about whether teachers can adapt to this technological wave but also about how they can transform AI technology into a powerful tool to enhance teaching quality and research efficiency. In this context, teachers’ generative artificial intelligence literacy may become one of the key factors influencing their sense of professional flourishing. The effective use of AI can not only alleviate teachers’ workloads and improve teaching efficiency but also bring more innovation and a sense of achievement to teachers. It can redefine their roles in teaching and research, thereby promoting the enhancement of their professional flourishing. However, existing literature has only focused on factors such as job satisfaction (Hakanen et al., 2006), achievement (Schaufeli et al., 2009), self-efficacy, professional identity (Kurrle and Warwas, 2025), work engagement, burnout, social support, and organizational climate in relation to teachers’ sense of professional flourishing. These studies have primarily explored how external work conditions and personal characteristics influence teachers’ wellbeing and professional development.

From the existing literature, it can be observed that there are several limitations in research on teachers’ professional flourishing. First, existing studies have primarily focused on traditional dimensions such as job satisfaction, career achievement, and mental health, with limited attention given to emerging factors like generative artificial intelligence literacy. As generative AI technology becomes more prevalent in the field of education, teachers need not only traditional teaching and research skills but also the ability to use AI tools to enhance teaching efficiency and research output. However, existing studies have not explored in depth how generative AI literacy impacts teachers’ professional flourishing. Second, most studies have not sufficiently considered the role of external support and resources, such as emotional support from the school, tool support, and academic atmosphere, all of which could have significant effects on teachers’ professional flourishing. Finally, research on teachers’ professional flourishing in the current literature often lacks a comprehensive perspective and has not systematically integrated multiple influencing factors. Therefore, this study aims to fill this gap by examining how personal ability factors such as generative AI literacy, along with various external support factors, collectively impact teachers’ professional flourishing.

The research questions addressed in this study are: First, what factors influence teachers’ sense of professional flourishing? Second, how does teachers’ generative AI literacy affect their sense of professional flourishing?

## 2 Literature review

### 2.1 Theoretical foundation

In educational research, professional flourishing is often viewed as a positive career state that reflects not only teachers’ mental health and work status but also their perception of the meaning of their profession and their pursuit of professional growth. Professional flourishing represents the intrinsic motivation, professional development, and sense of belonging that teachers experience in their work. These elements collectively form teachers’ subjective judgment of career wellbeing. Teachers’ sense of professional flourishing is closely linked not only to their intrinsic motivation but also to the strong influence of their work environment and external support (Kurrle and Warwas, 2025). Therefore, this study uses *self-determination theory* and *social support theory* as its theoretical foundation.

Self-determination theory, proposed by Deci and Ryan (1985), focuses on the basic needs related to behavior motivation and psychological wellbeing. The theory suggests that an individual’s intrinsic motivation and professional wellbeing are influenced by three core needs such as autonomy, competence, and relatedness. Autonomy refers to the ability to make independent choices and control one’s actions, while competence reflects the sense of effectiveness and self-improvement experienced when performing tasks. Relatedness represents the need to establish positive relationships with others. According to self-determination theory, when these needs are fulfilled, an individual’s intrinsic motivation and job satisfaction significantly improve (Ryan and Deci, 2000), which also contributes to the formation of professional thriving. For teachers, fulfilling these needs in teaching and research processes enhances their sense of professional achievement and work engagement. Autonomy allows teachers to make personal choices in teaching methods and research directions, which enhances their job satisfaction and creativity. Competence helps teachers maintain a positive attitude when facing challenges and provides a sense of accomplishment through continuous improvements in teaching and research outcomes. Relatedness encourages teachers to establish positive relationships with colleagues, students, and the academic community, fostering teamwork and a sense of support at work, which ultimately increases motivation and job satisfaction.

Social support theory, proposed by Cohen and Wills (1985), emphasizes the crucial role of social support in individual job performance and psychological wellbeing. The theory suggests that social support factors, such as emotional support, instrumental support, and informational support, can significantly reduce work-related stress and enhance an individual’s ability to cope with work challenges. In the context of teachers’ careers, emotional and instrumental support provided by the organization has a significant impact on their professional thriving. Emotional support primarily comes from colleagues, leaders, and students, offering care and encouragement, which helps teachers alleviate psychological stress and improve emotional wellbeing. Instrumental support includes the resources and tools provided by educational organizations, such as teaching equipment, technological platforms, and research resources. Adequate instrumental support enhances teachers’ work efficiency and helps them achieve better outcomes in teaching and research activities. Therefore, teachers working in a supportive social environment experience higher levels of job satisfaction and achievement (Bakker and Demerouti, 2007).

**Figure 1:**
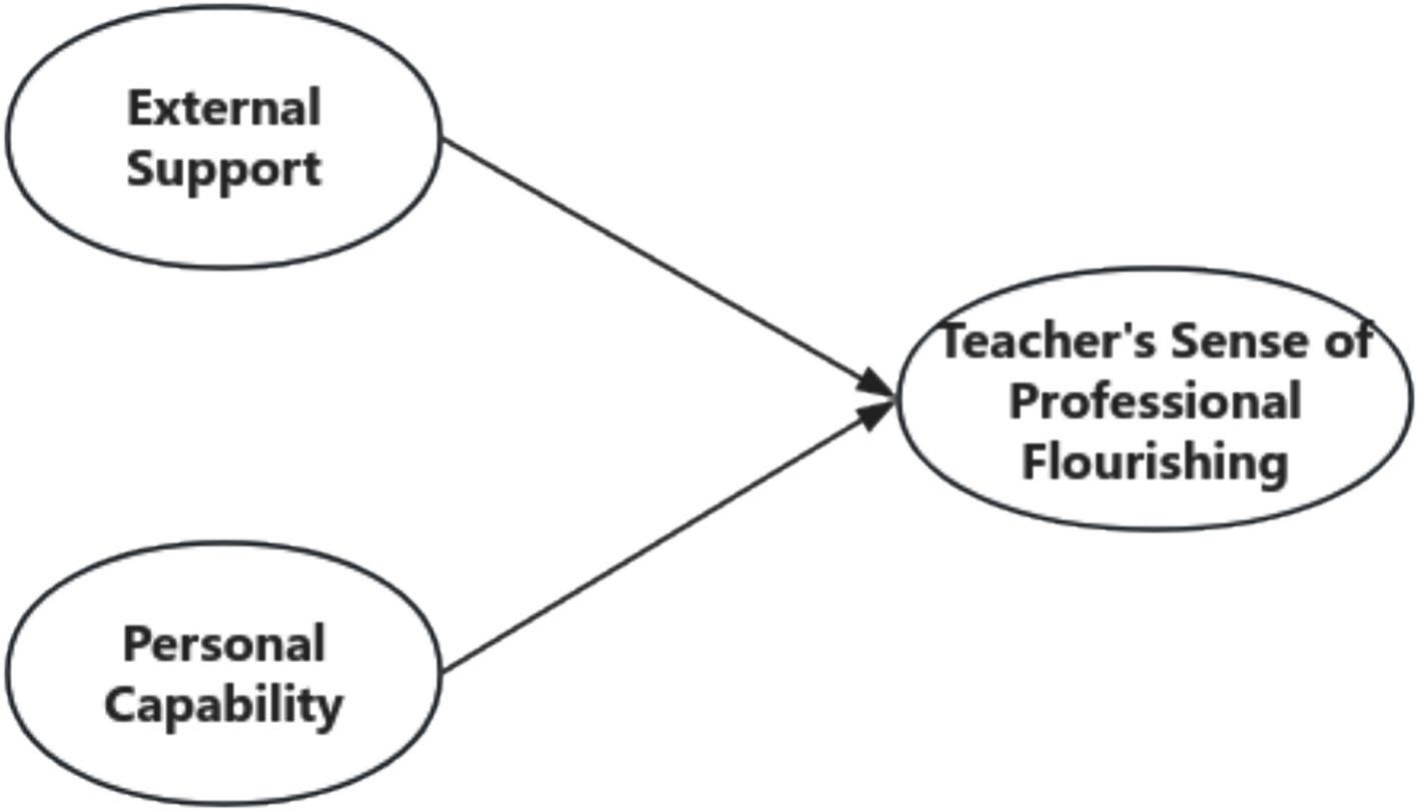
Multidimensional framework of teacher’s sense of professional flourishing.

Based on self-determination theory and social support theory, this study constructs a theoretical model called the “Multidimensional Framework of Teacher’s Sense of Professional Flourishing.” It examines the formation mechanism of teacher professional thriving from two dimensions, external support and personal capability (Figure 1).

**Figure 2:**
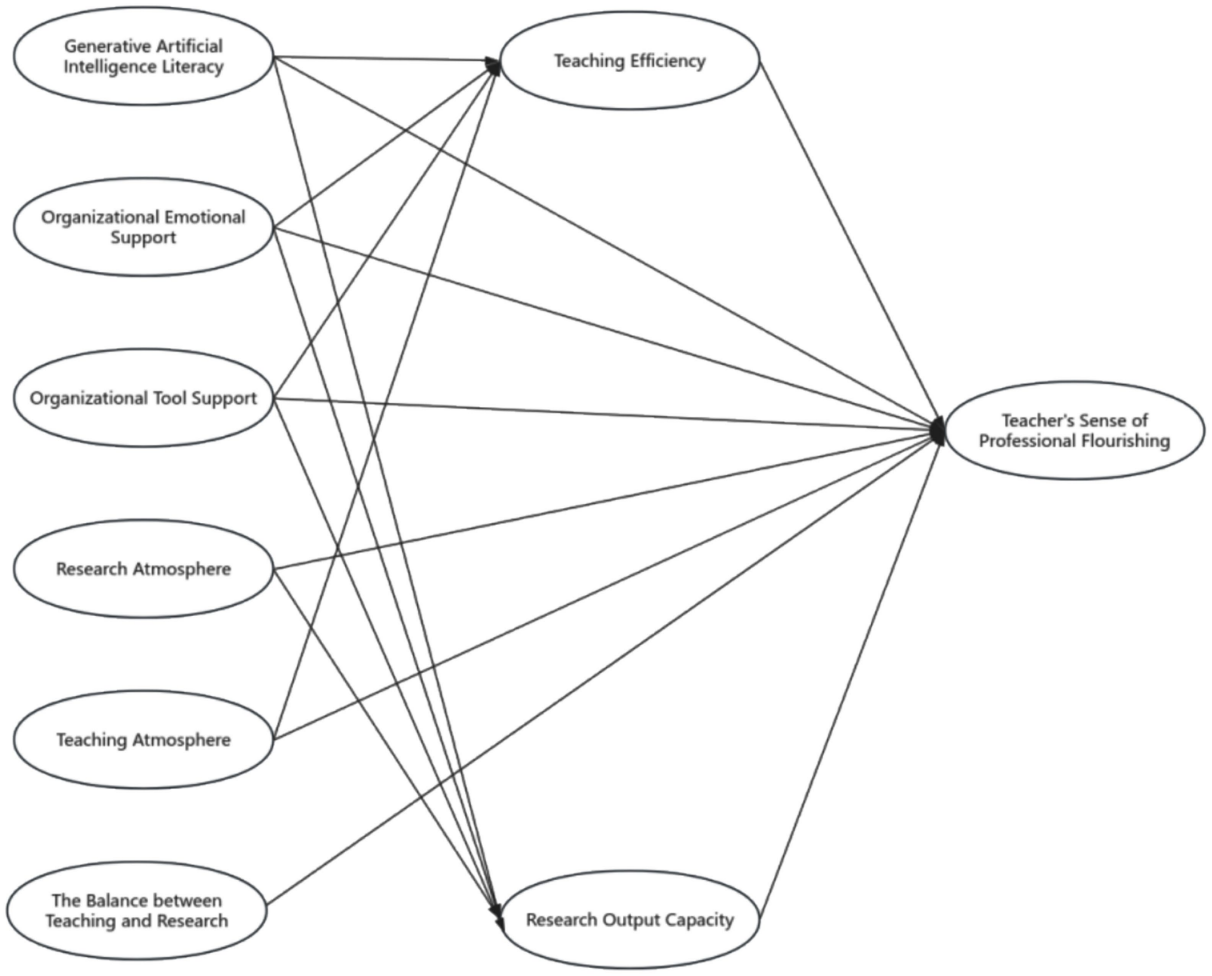
Research model.

**Figure 3:**
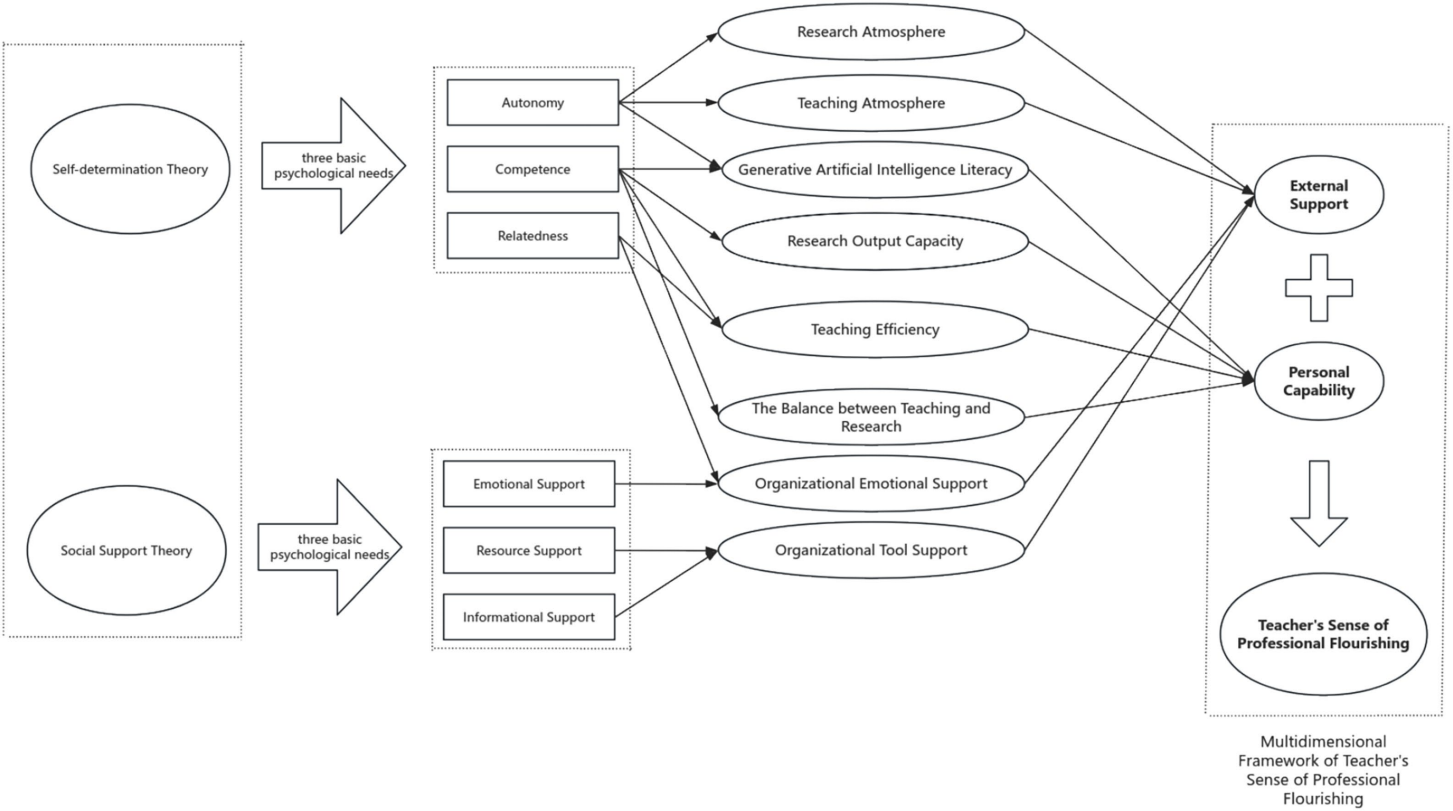
Logical chain for selecting the elements in the research model.

When constructing the research model (Figure 2) based on the theoretical framework (Figure 1) and selecting the elements within the research model, the choices were made based on the three basic psychological needs of *self-determination theory* (autonomy, competence, and relatedness) and the framework of emotional support, resource support, and informational support from *social support theory*. The specific logical chain is illustrated in Figure 3.

*The external support dimension* includes organizational and academic support that teachers receive in their work environment. According to self-determination theory, external support plays a crucial role in meeting teachers’ basic psychological needs, thus influencing their professional flourishing. The core argument of self-determination theory is that teachers’ professional flourishing is closely related to the fulfillment of their needs for autonomy, competence, and relatedness. Therefore, the variables in the external support dimension can directly affect these basic needs and subsequently impact professional flourishing.

Organizational emotional support corresponds closely with the need for relatedness. According to social support theory, emotional support refers to the care, understanding, and encouragement teachers receive from the organization, colleagues, and leaders. This support helps alleviate work-related stress, improve emotional health, and strengthen teachers’ sense of belonging and job satisfaction. According to self-determination theory, when teachers feel supported and accepted, their need for relatedness is fulfilled, which enhances their intrinsic motivation and promotes professional flourishing. Organizational tool support is linked to the need for competence. This refers to the resources, tools, and technology platforms provided to teachers during their teaching and research, such as teaching equipment, research resources, and technological support. According to social support theory, resource support helps teachers improve their work efficiency and effectiveness. In the framework of self-determination theory, competence is one of the core needs, and when teachers have the necessary tools and resources to perform their tasks, they feel more competent, which boosts their professional flourishing. Research atmosphere and teaching atmosphere are closely related to the need for autonomy. Research atmosphere reflects the academic resources and exchange opportunities provided by the school or organization. A positive research atmosphere stimulates teachers’ academic potential and fosters a sense of autonomy, as it provides teachers with more opportunities to engage in academic activities. According to self-determination theory, when teachers have more autonomy in their academic work, their intrinsic motivation is enhanced, leading to higher professional flourishing. Similarly, teaching atmosphere refers to the supportive nature of classroom interactions and the overall learning environment. A positive teaching atmosphere enables teachers to feel a sense of control over their teaching, which contributes to greater autonomy and a stronger sense of professional flourishing.

Teaching efficiency is another key element in the external support dimension. Teaching efficiency is closely related to competence, as it reflects how well teachers can manage their time, resources, and teaching strategies to achieve their goals. Self-determination theory suggests that when teachers are able to effectively manage their work and achieve high performance, their competence is enhanced, which positively influences their professional flourishing. Moreover, social support theory also plays a role here, as external resources, such as training, tools, and support from colleagues, can help teachers improve their efficiency and reduce work-related stress, further contributing to their professional flourishing.

*The personal capability dimension* focuses on teachers’ intrinsic abilities and technological literacy, including work-life balance and generative artificial intelligence literacy. According to self-determination theory, teachers’ professional flourishing is closely linked to their ability to balance their teaching and research responsibilities. A strong ability to manage this balance reduces work-related stress and prevents burnout, which enhances professional flourishing. Generative artificial intelligence literacy refers to teachers’ proficiency in using modern educational technologies. As generative AI becomes increasingly prevalent in education, teachers with high technological literacy can improve teaching efficiency, optimize lesson design, and promote research innovation, all of which contribute to their professional achievement and professional flourishing.

In summary, each variable in the external support dimension, such as organizational emotional support, organizational tool support, teaching efficiency, research atmosphere, and teaching atmosphere, is closely related to the three basic psychological needs of self-determination theory (relatedness, competence, and autonomy). Each variable helps meet these needs, thus promoting the enhancement of professional flourishing. At the same time, variables in the personal capability dimension, such as work-life balance and generative artificial intelligence literacy, contribute to enhancing teachers’ intrinsic motivation, competence, and autonomy, further supporting their professional flourishing. This makes the model theoretically grounded and closely connected to the core concepts of self-determination theory and social support theory.

### 2.2 Generative artificial intelligence literacy

With the intelligent transformation of the educational environment, teachers’ generative artificial intelligence (AI) literacy has become a significant driving force for their professional growth and development. According to existing research, teachers’ AI literacy can significantly enhance core dimensions of their professional flourishing, such as autonomy, competence, and relatedness (Huang and Zhao, 2025). These dimensions are key elements of self-determination theory, which suggests that when an individual’s basic psychological needs are fulfilled, their intrinsic motivation and job satisfaction improve significantly. Specifically, teachers with higher AI literacy are better able to independently select and control the use of AI tools, enhancing their effectiveness and creativity in teaching and research, thereby promoting work engagement, reducing stress, and increasing their sense of professional achievement.

From the perspective of social support theory, generative AI literacy is also closely related to the external support that teachers receive. Research indicates that teachers who have access to sufficient technological resources and support are better able to use AI tools for teaching innovation and research exploration (Bilbao-Eraña and Arroyo-Sagasta, 2025). The technological platforms, teaching resources, and organizational support provided by schools equip teachers with the necessary tools, enabling them to use AI more efficiently in teaching and research, thereby increasing their job satisfaction and work efficiency. At the same time, research shows that teachers’ ability to apply and innovate in a generative AI environment not only promotes their professional development at the cognitive level but also helps enhance their professional identity and sense of belonging, ultimately driving the improvement of their professional flourishing (Traga Philippakos and Rocconi, 2025). Studies have shown that teachers with higher AI literacy tend to perform more confidently and efficiently in classroom teaching, curriculum design, and technology integration (Bilbao-Eraña and Arroyo-Sagasta, 2025). Specifically, AI literacy helps improve teachers’ attitudes toward AI tools, strengthening their understanding and trust in educational technology. This positive attitude translates into more proactive use of AI tools in teaching, thus improving teaching efficiency and promoting teaching innovation (OECD, 2024). Furthermore, AI literacy enhances teachers’ technical application skills in teaching, enabling them to integrate modern technology more effectively, increasing classroom interaction and student participation.

In addition to directly improving teaching outcomes, AI literacy also promotes teachers’ professional development, particularly in terms of their adaptability within the intelligent education environment. As educational technology continues to evolve, teachers with higher AI literacy are better equipped to handle changes and challenges in the educational environment, enhancing their professional competitiveness and adaptability (Traga Philippakos and Rocconi, 2025). This enhanced adaptability not only helps teachers work more efficiently within new educational models but also strengthens their sense of professional identity and belonging, thus further enhancing their professional flourishing. Based on the above literature analysis, the following hypothesis is proposed:

*H1*: The higher the teacher's generative artificial intelligence literacy, the stronger the teacher's sense of professional flourishing.

### 2.3 Organizational emotional support and organizational tool support

In recent years, teachers’ sense of professional flourishing has become an important topic in educational research, with scholars focusing on how external support systems can enhance teachers’ job satisfaction, sense of achievement, and mental wellbeing. In this context, organizational emotional support and organizational tool support are considered key factors. Organizational emotional support typically refers to the emotional care and psychological support teachers receive at work, while organizational tool support includes resources, technological platforms, and educational tools provided to teachers. Research has shown that these two types of support have a profound impact on teachers’ sense of professional flourishing (Bakker and Demerouti, 2007).

First, organizational emotional support plays a positive role in enhancing teachers’ sense of professional flourishing. Emotional support comes from the care and support provided by school leaders, colleagues, and students. This support effectively alleviates teachers’ job-related stress and promotes their emotional wellbeing (Bakker and Demerouti, 2007). Emotional support also fosters a sense of belonging and self-identity, making teachers feel valued and recognized in their careers, which strengthens their commitment to the education profession (Collie et al., 2012). Research has shown that when teachers receive emotional support, they are more confident in handling teaching challenges and maintain higher job satisfaction, directly contributing to the enhancement of professional flourishing (Hakanen et al., 2006). In the teacher’s work environment, the emotional support provided by the organization creates a safe psychological space, helping teachers buffer emotional stress when facing high work pressure, thereby improving their job satisfaction and mental health.

Second, organizational tool support also has a significant impact on teachers’ sense of professional flourishing. Tool support refers to the resources and equipment provided by the organization, such as teaching materials, technology platforms, and course design tools. Sufficient tool support can significantly enhance teachers’ work efficiency and teaching effectiveness, thereby increasing their sense of professional achievement. In modern educational environments, teachers’ reliance on technology platforms has become stronger, and educational technology support provided by the organization helps teachers perform better in both classroom teaching and research. For example, digital technology platforms and online teaching tools can reduce teaching pressure, improve classroom interaction, and increase student engagement, which enhances teachers’ job satisfaction and sense of achievement (Zhang et al., 2025).

Furthermore, the combination of emotional support and tool support can form a supportive system that promotes an overall improvement in teachers’ professional flourishing. Research shows that when teachers receive adequate emotional and tool support, their job satisfaction, teaching engagement, and professional identity are significantly enhanced. This comprehensive support helps teachers maintain a positive professional attitude in complex teaching environments (Hidayati et al., 2025). Based on the above literature analysis, the following hypotheses are proposed:

*H2*: The stronger the organizational emotional support received by teachers, the stronger their sense of professional flourishing.

*H3:* The stronger the organizational tool support received by teachers, the stronger their sense of professional flourishing.

### 2.4 Research atmosphere and teaching atmosphere

Scholars have increasingly focused on the impact of the work environment on teachers’ sense of professional flourishing, particularly the roles played by the research atmosphere and the teaching atmosphere. The research atmosphere refers to the supportive and encouraging environment that teachers experience within the academic setting, which typically includes opportunities for academic exchange, research resource support, and collaboration among colleagues. A positive research atmosphere can stimulate teachers’ academic interest and innovative capabilities, thereby enhancing their sense of professional achievement. Existing studies have shown that when teachers work in a supportive research atmosphere, they receive more academic support and collaboration opportunities, which not only help improve their academic accomplishments but also enhance their professional identity and sense of belonging, ultimately boosting their professional flourishing (Hakanen et al., 2006).

Moreover, the resource support and collaboration opportunities within the research atmosphere can help reduce teachers’ burnout, enhance their work motivation, and improve their sense of self-efficacy and professional wellbeing (Bakker and Demerouti, 2007). Research has also indicated that a positive work climate in schools or educational institutions significantly influences teachers’ professional wellbeing and job satisfaction. When teachers perceive a supportive work environment from the organization, their job satisfaction, professional commitment, and psychological wellbeing increase significantly, suggesting that working in a supportive academic environment contributes to greater professional engagement and wellbeing (Dreer, 2022). Furthermore, supportive factors in the teaching and research environment help improve teachers’ professional confidence and work engagement, which in turn strengthens their professional identity and motivation for continuous development. These factors provide essential conditions for achieving a higher level of professional flourishing (Zhao, 2022). Therefore, the supportive atmosphere in the work environment, both in terms of research and teaching, plays a key role in enhancing teachers’ sense of professional flourishing.

The teaching atmosphere refers to the work environment that teachers experience during classroom teaching, including factors such as student feedback, support from colleagues, and the provision of teaching resources. The teaching atmosphere directly influences teachers’ teaching engagement, teaching effectiveness, and professional identity. Studies have shown that a positive teaching atmosphere helps teachers maintain an optimistic work attitude, boosting their teaching motivation and confidence. Teachers in a supportive and positive teaching environment can better achieve teaching goals, improve teaching outcomes, and increase job satisfaction (Collie et al., 2012). Moreover, the teaching atmosphere also indirectly impacts teachers’ sense of professional flourishing by enhancing their teaching confidence and job satisfaction, which further strengthens their sense of achievement and belonging.

Additionally, the interaction between the research atmosphere and the teaching atmosphere should not be overlooked. Teachers in an environment that offers both strong research support and a positive teaching atmosphere are able to develop academic and teaching abilities more effectively. This environment fosters overall professional development. Research has found that the positive interaction between research and teaching can strengthen teachers’ professional confidence and sense of achievement, thereby enhancing their sense of professional flourishing (Fernández-Batanero et al., 2021). In summary, both the research atmosphere and the teaching atmosphere have a profound impact on teachers’ sense of professional flourishing. A positive research atmosphere promotes teachers’ academic development and innovation, increasing their sense of professional achievement. A positive teaching atmosphere enhances teaching motivation and job satisfaction, strengthening professional identity and belonging. Their interaction ultimately helps teachers maintain a high level of professional flourishing in complex educational environments. Based on the above analysis, the following hypotheses are proposed:

*H4*: The better the research atmosphere, the stronger the teacher's sense of professional flourishing.

*H5*: The better the teaching atmosphere, the stronger the teacher's sense of professional flourishing.

### 2.5 The balance between teaching and research

The ability of teachers to balance work and life between teaching and research has a significant impact on their sense of professional flourishing. First, the ability to balance work and life helps teachers effectively allocate time and energy, preventing the excessive accumulation of work pressure. By finding a balance between these two important tasks, teachers can reduce their psychological burden and job burnout, thus improving their job satisfaction and mental health (Wei and Ye, 2022). When teachers are able to manage their time effectively, dedicating time to both teaching and research, their sense of accomplishment and work motivation are enhanced, which directly contributes to the improvement of their professional flourishing.

Second, a good work-life balance enhances teachers’ sense of self-efficacy. When teachers achieve a balance between teaching and research, they can maintain high work performance and creativity, thereby improving their academic and teaching outcomes. This increase in self-efficacy allows teachers to feel a stronger sense of professional value, which in turn boosts their professional identity and sense of belonging (Hakanen et al., 2006). This sense of identity and belonging not only contributes to teachers’ professional growth but also strengthens their long-term commitment to the education profession, further enhancing their sense of professional flourishing. Moreover, the ability to balance work and life also enhances teachers’ emotional health and mental wellbeing, which in turn improves their sense of professional flourishing. In an environment where work and personal life are balanced, teachers are able to maintain greater emotional stability and psychological health, which helps foster a more positive attitude and greater work engagement. Studies show that when teachers feel their work and personal life are in balance, their happiness, work motivation, and professional commitment significantly increase (Vo et al., 2024), which further drives the development of their professional flourishing. Based on the above analysis, this study proposes the following hypothesis:

*H6*: The stronger the ability of work-life balance between teaching and research, the stronger the teacher's sense of professional flourishing.

### 2.6 Teaching efficiency and research output capacity

Research has shown that teaching efficiency and research output capacity significantly influence teachers’ sense of professional flourishing. First, improving teaching efficiency can effectively reduce teachers’ work-related stress, enhance their self-efficacy, and increase job satisfaction, thereby boosting their professional flourishing. Specifically, when teachers are able to complete teaching tasks efficiently, they often experience a greater sense of achievement and professional identity in their teaching (Kurrle and Warwas, 2025). This not only contributes to improved student learning outcomes but also enhances the teachers’ own sense of professional accomplishment (Bakker and Demerouti, 2007). Additionally, efficient teaching allows teachers to allocate more time to research and personal life, further improving their work-life balance, and thus enhancing their overall professional wellbeing (Schaufeli et al., 2009).

Regarding research output capacity, teachers who produce more academic results through research activities not only enhance their academic reputation but also gain more career development opportunities. Studies have indicated that teachers’ research output capacity is closely related to their sense of professional identity and belonging, particularly in higher education environments. Successful research output can significantly increase teachers’ job satisfaction and work motivation (Hakanen et al., 2006). Teachers with higher research output are often recognized more in the academic field, providing them with more opportunities for promotion and research funding, further strengthening their sense of professional achievement and flourishing (Bakker and Demerouti, 2007). Based on the above literature analysis, the following hypotheses are proposed:

*H7*: The higher the teaching efficiency, the stronger the teacher's sense of professional flourishing.

*H8*: The stronger the research output capacity, the stronger the teacher's sense of professional flourishing.

The research model of this study is shown in Figure 2.

## 3 Methods

### 3.1 Data source

The subjects of this study are university professors from ordinary universities (non-key public universities) in Jinan, Shandong Province, with a focus on their sense of professional flourishing. To ensure the representativeness and comprehensiveness of the survey, this study used a stratified random sampling method to select several ordinary universities in Jinan as the survey targets, covering a range of disciplines and teaching positions, thus ensuring both breadth and representativeness. First, Jinan has a total of 10 districts, but only six districts have universities, while the other non-administrative areas do not have universities. A total of 90 questionnaires were distributed in each of the six districts. Second, within these six districts, two universities were randomly selected by drawing lots in each district, with 45 questionnaires distributed to each university. Third, the professors in each university were divided into two types: natural science professors and social science professors. Twenty-three questionnaires were distributed to natural science professors, while 22 questionnaires were distributed to social science professors. Finally, within each discipline, the questionnaires were distributed proportionally in a 1:1 male-to-female ratio.

From July 5 to August 1, 2025, the research team distributed electronic questionnaires to 540 university professors in Jinan. To ensure the validity and representativeness of the data, strict screening criteria were applied. These criteria included: only full-time faculty members from universities in Jinan were eligible to participate, and all questions related to professional flourishing had to be fully completed. Furthermore, to guarantee the quality of the data, the completeness, logical consistency, and reasonableness of the responses were carefully reviewed. Incomplete or logically inconsistent answers were excluded. After the screening process, 540 valid questionnaires were returned. After removing low-quality responses, the remaining 499 valid questionnaires provided a comprehensive representation of the sense of professional flourishing among university professors in Jinan.

Regarding ethical considerations, since the author’s research institution does not have an ethics review board, the study was authorized and approved by the department chair or relevant leadership. Participants were fully informed about the purpose of the study, the voluntary nature of their participation, and the confidentiality of their responses. Informed consent was obtained from all participants, ensuring their anonymity throughout the research process. All data collected was used solely for academic purposes.

### 3.2 Scale design and variable development

The teacher professional flourishing scale is adapted from existing well-established scales and aims to assess professional flourishing and teacher wellbeing. The items in the scale are based on dimensions used in prior literature, such as career achievement, job satisfaction, and professional growth. These dimensions have been proven to be effective indicators of teacher professional flourishing (e.g., Hakanen et al., 2006; Ji et al., 2009). The Teacher Professional Flourishing scale consists of five items designed to assess teachers’ perceptions of their professional experiences. These items include: (1) the extent to which teachers feel proud of their teaching achievements, (2) the meaning they attribute to their career, (3) the positive impact they believe their work has on students and society, (4) the sense of fulfillment derived from their work, and (5) whether they feel they have experienced ongoing growth and progress in their career. The scale uses a 5-point Likert scale, ranging from “strongly disagree” to “strongly agree.”

The Generative Artificial Intelligence Literacy scale is adapted from an existing scale (Zhong and Liu, 2025) and consists of five items that assess teachers’ understanding and application of generative artificial intelligence (AI) in their professional work. The items include evaluating how well teachers understand the use of generative AI, their ability to apply AI tools in teaching, and their capacity to assist students in utilizing generative AI for personalized learning. Additionally, the scale assesses how teachers can use generative AI to improve their teaching content and methods, as well as their perceptions of the positive impact of generative AI on the future of education. The scale uses a 5-point Likert scale, ranging from “strongly disagree” to “strongly agree,” to capture teachers’ generative AI literacy.

The Organizational Emotional Support scale is adapted from existing scales (Bakker and Demerouti, 2007; Collie et al., 2012) used to measure emotional support in organizational contexts, particularly in educational settings. This scale includes items that assess the extent to which teachers feel that their emotional needs are recognized and addressed by school leadership and colleagues. The items include: the extent to which teachers feel that school leadership cares about their emotional needs, the support for teachers’ emotional health in the work environment, the emotional support provided by the school in times of work-related stress, the understanding and care shown by school leadership and colleagues when teachers face difficulties, and the overall satisfaction with the fulfillment of emotional needs within the school.

The Organizational Tool Support scale is adapted from existing measures in the literature that assess the availability of resources and tools in the work environment and their impact on employee outcomes. This scale is designed to measure the extent to which teachers perceive that their institution provides sufficient resources and tools to support their teaching and research activities. The items include the degree to which teachers can obtain adequate teaching resources (such as textbooks and instructional materials) to support their work, the extent to which the technology platforms provided by the school (such as online teaching platforms and digital tools) are helpful for instruction, how easily teachers can access necessary teaching equipment (such as computers and projectors), the adequacy of resources provided for their research work (such as research funding and laboratory equipment), and whether the tools and resources available help enhance their capabilities in teaching and research. All items are measured on a 5-point Likert scale ranging from “strongly disagree” to “strongly agree.” The selection of these items is supported by research demonstrating that organizational resources and tools function as job resources that help reduce strain and improve work performance. For example, the job demands-resources model highlights the role of organizational resources, including access to tools and equipment, in enhancing employee engagement and wellbeing (Bakker and Demerouti, 2007). In educational contexts, research has shown that perceived resource adequacy, including access to instructional materials and professional supports, is positively related to teachers’ sense of efficacy and job satisfaction (Skaalvik and Skaalvik, 2014).

The Research Atmosphere scale includes five items, adapted from an existing scale (Sibanda, 2021), and is designed to measure the level of support that teachers perceive in the research environment. The items include: (1) whether the school provides opportunities for teachers to engage in academic discussions; (2) whether the research atmosphere at the school encourages teachers to conduct more academic research; (3) whether the school encourages research collaboration and academic exchanges; (4) whether the school provides necessary support and resources for research activities; and (5) whether the research atmosphere at the school helps to improve teachers’ research capabilities.

A positive Teaching Atmosphere can significantly enhance teachers’ teaching efficiency and innovation. The Teaching Atmosphere scale also includes five items, adapted from an existing scale (Rodríguez-Izquierdo et al., 2020), and is designed to assess the level of support that teachers perceive in the teaching environment. The items include: (1) whether the school provides a positive teaching atmosphere to support teachers’ work; (2) whether the school encourages innovative teaching methods; (3) the level of student engagement; (4) whether the school provides resources and opportunities for teaching innovation; and (5) whether the school supports teachers’ teaching practices and methods. The support provided by a positive teaching atmosphere can enhance the quality of teaching and teaching satisfaction, thereby promoting teachers’ sense of professional flourishing.

The Teaching-Research Balance scale includes five items, adapted from an existing scale (Greenhaus and Allen, 2011), to assess teachers’ ability to balance their teaching and research responsibilities. These items are as follows: (1) To what extent are you able to find a good balance between teaching and research? (2) To what extent are you able to allocate time reasonably to balance teaching and research tasks? (3) To what extent do you feel that balancing teaching and research contributes to your career development? (4) To what extent are you able to efficiently complete both teaching and research tasks at the same time? (5) To what extent do you feel that maintaining a balance between teaching and research helps reduce work-related stress?

The Teaching Efficiency and Research Output Capability scales are self-designed to assess teachers’ efficiency in teaching and research activities. The Teaching Efficiency scale includes five items: (1) To what extent can you efficiently prepare and complete teaching content for each class? (2) To what extent can you complete teaching tasks within the allocated time? (3) To what extent can you organize classroom activities to ensure student participation? (4) To what extent can you improve classroom efficiency through optimized teaching methods? (5) To what extent can you manage your time effectively to enhance teaching efficiency?

The Research Output Capability scale also includes five items: (1) To what extent can you complete and submit high-quality research papers within the given time? (2) To what extent can you design and implement research projects effectively? (3) To what extent can you maintain consistent research output (e.g., papers, monographs)? (4) To what extent can you collaborate with peers to increase research output? (5) To what extent can you access sufficient research resources to support your projects?

In this study, all scales used a 5-point Likert scale, ranging from “strongly disagree” to “strongly agree.” Additionally, the questionnaire included several attention-check questions to assess respondents’ engagement and attentiveness during the survey. These questions were designed to ensure the quality and reliability of the responses. A personal information module was also included to gather demographic data, such as gender, age, income level, and the frequency of using generative artificial intelligence tools. This module helps to provide context for the survey results and allows for a more nuanced analysis of the factors influencing teacher professional flourishing, teaching efficiency, and other related variables.

### 3.3 Sample information

The sample consists of 499 participants, with a near-equal gender distribution (49.9% male and 50.1% female). Age-wise, the majority fall within the 21–30 and 41–50 age groups, accounting for 26.8 and 26.4%, respectively. In terms of income, most participants earn between 4,001 and 6,000 RMB (19.64%) and 8,001–10,000 RMB (21.24%), followed by the 6,001–8,000 RMB (18.64%) group. The income distribution shows a relatively balanced spread across various brackets, with fewer participants in the higher income ranges (Table 1).

**Table 1:** Sample information.

Item	Option	Frequency	Percentage (%)
GENDER	Male	249	49.9
	Female	250	50.1
AGE	21–30	133	26.8
	31–40	124	24.8
	41–50	132	26.4
	51–60	87	17.4
	Above 60	23	4.6
INCOME	2001–4,000 RMB	42	8.41
	4,001–6,000 RMB	98	19.64
	6,001–8,000 RMB	93	18.64
	8,001–10,000 RMB	106	21.24
	10,001–15,000 RMB	98	19.64
	15,001–20,000 RMB	37	7.41
	Above 20,000 RMB	25	5.01
Total		499	100.0

### 3.4 Descriptive statistics

The statistical results (Table 2) of basic indicators show that the scores of all core variables follow a normal distribution trend. The skewness of most indicators is negative, indicating that most respondents rated the variables at the higher end of the scale. The kurtosis values indicate that the distribution of most indicators is concentrated and close to normal, with few extreme ratings. Regarding the sample size, all core variables were represented by 499 samples, ensuring the representativeness and reliability of the data, and effectively reflecting the overall situation of the teacher group. The minimum and maximum values in the data represent the lowest and highest ratings for each dimension. For example, in the GAI dimension, the minimum value was 4.0 and the maximum value was 5.0, showing that most respondents’ evaluations were consistent and positive. In terms of average values and standard deviations, most core variables had an average score close to or above 4.0, indicating that teachers generally held a positive attitude toward these dimensions. However, some variables, such as OES1 (organizational emotional support) and BTR1 (teaching and research balance), had larger standard deviations, suggesting considerable differences in respondents’ perceptions. This may be related to individual career backgrounds, work environments, or personal experiences.

**Table 2:** Descriptive statistics.

	Name	Variance	S. E	IQR	Kurtosis	Skewness	Coefficient of variation (CV)
Teacher’s Sense of Professional Flourishing (TSPF)	TSPF1	0.294	0.024	0.000	3.617	−0.516	13.073%
	TSPF2	0.360	0.027	1.000	1.973	−1.278	13.170%
	TSPF3	0.371	0.027	1.000	1.470	−0.649	14.059%
	TSPF4	0.400	0.028	1.000	2.490	−0.795	14.857%
	TSPF5	0.394	0.028	1.000	1.345	−0.806	14.312%
Generative Artificial Intelligence Literacy (GAI)	GAI1	0.307	0.025	1.000	−0.207	−0.100	12.852%
	GAI2	0.474	0.031	1.000	1.680	−0.858	16.194%
	GAI3	0.520	0.032	1.000	0.954	−0.692	17.363%
	GAI4	0.444	0.030	1.000	1.013	−0.801	15.494%
	GAI5	0.424	0.029	1.000	0.723	−0.716	14.980%
Organizational Emotional Support (OES)	OES1	0.625	0.035	1.000	0.629	−0.620	21.158%
	OES2	0.677	0.037	1.000	1.459	−0.963	20.253%
	OES3	0.843	0.041	2.000	0.258	−0.732	23.659%
	OES4	0.589	0.034	1.000	0.337	−0.691	18.692%
	OES5	0.755	0.039	1.000	0.403	−0.731	22.060%
Organizational Tool Support (OTS)	OTS1	0.389	0.028	1.000	1.520	−0.717	14.417%
	OTS2	0.425	0.029	1.000	1.599	−0.864	15.025%
	OTS3	0.434	0.029	1.000	1.326	−0.869	15.131%
	OTS4	0.670	0.037	1.000	0.635	−0.669	21.004%
	OTS5	0.483	0.031	1.000	1.949	−0.885	16.585%
Research Atmosphere (RA)	RA1	0.466	0.031	1.000	1.777	−0.747	16.620%
	RA2	0.651	0.036	1.000	1.015	−0.848	19.688%
	RA3	0.557	0.033	1.000	1.251	−0.862	17.907%
	RA4	0.598	0.035	1.000	1.033	−0.853	18.732%
	RA5	0.579	0.034	1.000	1.740	−0.994	18.351%
Teaching Atmosphere (TA)	TA1	0.449	0.030	1.000	2.250	−0.918	15.737%
	TA2	0.521	0.032	1.000	1.610	−0.981	16.884%
	TA3	0.554	0.033	1.000	1.502	−0.842	18.141%
	TA4	0.545	0.033	1.000	1.122	−0.769	17.913%
	TA5	0.472	0.031	1.000	0.909	−0.685	16.291%
Balance in Teaching and Research Capacity (BTR)	BTR1	0.488	0.031	0.000	1.335	−0.718	17.437%
	BTR2	0.607	0.035	1.000	1.046	−0.812	19.008%
	BTR3	0.438	0.030	1.000	1.092	−0.725	15.436%
	BTR4	0.628	0.035	1.000	0.979	−0.795	19.769%
	BTR5	0.754	0.039	1.000	0.394	−0.793	21.560%
Teaching Efficiency (TE)	TE1	0.335	0.026	1.000	−0.277	−0.237	13.391%
	TE2	0.332	0.026	1.000	−0.351	−0.424	13.043%
	TE3	0.433	0.029	1.000	0.916	−0.533	15.795%
	TE4	0.410	0.029	1.000	0.207	−0.510	14.969%
	TE5	0.396	0.028	1.000	−0.175	−0.370	14.752%
Research Output Capacity (ROC)	ROC1	0.595	0.035	0.000	0.287	−0.541	19.468%
	ROC2	0.554	0.033	1.000	1.196	−0.720	18.397%
	ROC3	0.757	0.039	1.000	−0.235	−0.455	22.551%
	ROC4	0.612	0.035	1.000	0.839	−0.825	18.912%
	ROC5	0.715	0.038	1.000	0.644	−0.778	21.199%

The median value for most variables was 4.0 or 5.0, further confirming that teachers’ evaluations are mostly concentrated in the higher rating range. The interquartile range (IQR) was typically 1, indicating that most respondents’ ratings were clustered between the 25th and 75th percentiles, showing a certain degree of concentration. As for skewness, many variables displayed negative skewness (e.g., TSPF, GAI), meaning that most respondents’ ratings were higher. This indicates that most teachers’ evaluations of professional flourishing and generative AI literacy were positive, with fewer extreme low scores. Additionally, the kurtosis results show that the distribution of most indicators is concentrated and tends to follow a normal distribution. However, certain indicators, such as OES1 and BTR1, exhibited higher kurtosis, suggesting that the scores for these indicators are more concentrated with fewer extreme values. The coefficient of variation (CV) analysis reveals that many indicators had a low CV (e.g., GAI and TA), indicating that most teachers’ evaluations of these dimensions were consistent. However, certain indicators, such as OES1 and BTR1, showed a higher CV, suggesting significant differences in teachers’ evaluations of emotional support and teaching-research balance, which may be due to varying individual experiences in these areas.

### 3.5 Reliability and validity analysis

The Cronbach *α* coefficients (Table 3) indicate that OES (0.858) and ROC (0.798) have high reliability (above 0.8), effectively reflecting teachers’ perceptions of emotional support and research output. RA (0.795) and BTR (0.731) fall between 0.7 and 0.8, showing good reliability in measuring teachers’ perceptions of the research environment and their ability to balance teaching and research. TA has a Cronbach α of 0.719, indicating good reliability for measuring teaching environment perceptions. GAI (0.639), OTS (0.666), and TE (0.624) fall between 0.6 and 0.7, indicating acceptable reliability.

**Table 3:** Reliability analysis.

Item	Cronbach α
TSPF	0.679
GAI	0.639
OES	0.858
OTS	0.666
RA	0.795
TA	0.719
BTR	0.731
TE	0.624
ROC	0.798

According to the validity analysis results, the factor loading coefficients and communalities (common factor variance) were first analyzed. The factor loading coefficient reflects the correlation of each variable with different factors. A higher factor loading coefficient (with an absolute value greater than 0.4) indicates a strong relationship between the variable and the factor. The data show that most variables have high factor loading coefficients, particularly in Factor 1, where several variables (such as TSPF1, OES1, GAI1, etc.) have strong factor loadings, indicating a strong correlation of these variables with this factor. Next, communalities (common factor variance) reflect the shared variance of each variable with all factors. A higher communality value indicates that the variable is closely related to the factor and can effectively explain the characteristics of that factor. In this analysis, dimensions such as OES and TSPF have generally high communalities, indicating that these variables can effectively reflect the characteristics of the latent factors.

In the factor analysis rotation results, the KMO value was 0.947, indicating that the sample is suitable for factor analysis and that the results are applicable. The Bartlett’s test of sphericity yielded a *p*-value of 0.000, indicating significant correlations between the variables, thus allowing the continuation of factor analysis. The eigenvalues of the rotated factors showed that the first few factors had higher eigenvalues, with the explained variance also being high. Specifically, Factor 1, Factor 2, and Factor 3 explained 14.909, 10.284, and 4.853% of the variance, respectively. The cumulative explained variance after rotation reached 54.574%, indicating that factor analysis can adequately explain the variability in the data. Furthermore, to avoid the impact of common method bias (CMB) on the results, the study conducted a Harman single-factor test. The results showed that the variance explained by the first factor’s eigenvalue was 31.278%, which is below 50%, indicating that there is no common method bias issue in this study.

Overall, the communalities for all study items were above 0.4, indicating that the information for these items can be effectively extracted. Additionally, the KMO value was 0.947, greater than 0.6, meaning that data can be effectively extracted. The variance explained by the nine factors was 14.909, 10.284, 4.853, 4.550, 4.248, 4.049, 3.979, 3.906, and 3.796%, respectively. The cumulative variance explained after rotation was 54.574%, greater than 50%. This indicates that the information from the study items can be effectively extracted (Table 4).

**Table 4:** Validity analysis.

Item	Factor loading									Communality (common factor variance)
	Factor 1	Factor 2	Factor 3	Factor 4	Factor 5	Factor 6	Factor 7	Factor 8	Factor 9	
TSPF1	0.097	0.268	0.376	0.256	0.478	0.112	−0.034	0.171	−0.141	0.580
TSPF2	0.214	0.107	−0.063	0.656	0.117	0.083	0.191	0.100	0.175	0.590
TSPF3	0.139	0.066	0.119	0.217	0.547	−0.008	0.137	0.223	−0.012	0.452
TSPF4	0.045	0.302	0.109	0.399	0.350	0.086	0.019	0.171	0.164	0.451
TSPF5	0.153	0.144	0.163	0.712	0.150	0.010	0.157	0.011	−0.050	0.627
GAI1	0.094	0.042	0.684	−0.035	0.176	0.080	0.166	0.053	0.081	0.554
GAI2	0.111	0.087	0.220	0.138	0.003	0.099	0.609	0.047	−0.057	0.473
GAI3	0.292	0.107	0.369	0.164	−0.090	−0.145	0.368	0.215	−0.013	0.471
GAI4	0.253	0.055	0.610	0.089	0.114	0.066	0.221	−0.062	0.079	0.523
GAI5	0.140	0.160	0.068	0.130	0.103	0.060	0.713	0.037	0.081	0.598
OES1	0.320	0.727	0.096	0.024	0.144	0.132	0.115	0.004	−0.029	0.692
OES2	0.317	0.622	0.032	0.220	0.138	−0.028	0.058	0.132	0.139	0.597
OES3	0.330	0.669	0.084	0.062	0.156	0.073	0.173	0.080	0.030	0.634
OES4	0.266	0.589	0.022	0.170	0.034	0.150	0.085	0.024	0.211	0.523
OES5	0.336	0.709	0.073	0.072	0.101	0.065	0.138	0.086	0.049	0.670
OTS1	0.184	0.185	0.058	0.009	0.586	0.258	0.056	0.001	0.340	0.600
OTS2	0.427	0.122	0.030	0.310	−0.044	−0.063	0.071	0.185	0.380	0.483
OTS3	0.049	0.121	0.062	0.052	0.108	0.147	−0.005	0.017	0.706	0.556
OTS4	0.474	0.533	0.075	0.060	0.071	0.044	0.028	−0.011	0.083	0.533
OTS5	0.438	0.372	0.182	0.102	0.123	−0.111	0.127	0.093	0.302	0.517
RA1	0.491	0.222	0.346	0.243	0.226	0.194	−0.014	−0.218	0.057	0.609
RA2	0.602	0.268	0.090	0.292	0.051	−0.042	0.077	0.115	0.031	0.552
RA3	0.553	0.208	−0.091	0.218	0.229	0.164	0.103	0.005	0.167	0.523
RA4	0.585	0.258	0.155	0.235	−0.024	0.141	−0.031	0.022	0.087	0.517
RA5	0.576	0.318	0.098	0.047	0.177	−0.013	0.118	0.172	0.185	0.554
TA1	0.344	0.324	0.112	0.201	0.412	0.195	−0.019	0.002	0.223	0.534
TA2	0.569	0.109	0.117	0.125	0.136	−0.010	0.221	0.013	0.222	0.482
TA3	0.083	0.449	0.403	0.174	−0.044	0.056	0.023	0.247	0.253	0.532
TA4	0.455	0.291	0.154	0.188	0.183	0.089	0.026	0.050	0.304	0.488
TA5	0.459	0.284	0.129	0.039	0.157	−0.136	0.211	0.093	0.224	0.456
BTR1	0.483	0.260	0.334	0.044	−0.064	0.345	−0.098	0.084	−0.020	0.555
BTR2	0.562	0.210	0.050	−0.056	−0.065	0.116	0.045	0.371	0.027	0.524
BTR3	0.444	0.040	0.176	−0.075	0.259	0.025	0.194	0.080	0.281	0.426
BTR4	0.453	0.369	0.080	0.172	−0.084	0.410	0.013	0.092	−0.044	0.563
BTR5	0.429	0.281	0.210	−0.132	0.144	−0.051	0.080	0.391	−0.040	0.509
TE1	0.157	0.006	0.101	0.040	0.204	0.703	0.071	0.166	0.120	0.619
TE2	0.094	−0.020	−0.037	0.109	0.161	0.089	0.005	0.755	0.054	0.630
TE3	0.172	0.155	0.338	0.250	−0.266	0.337	0.047	0.212	0.316	0.562
TE4	0.098	0.239	0.047	0.001	0.141	0.536	0.436	0.079	0.164	0.599
TE5	0.174	0.226	0.131	0.098	0.073	0.215	0.147	0.515	0.077	0.452
ROC1	0.594	0.186	0.348	0.021	0.101	0.276	−0.088	0.068	−0.090	0.616
ROC2	0.562	0.202	0.017	0.090	0.035	0.231	0.181	0.273	−0.197	0.566
ROC3	0.598	0.314	0.075	−0.014	0.078	0.111	0.213	0.042	−0.110	0.539
ROC4	0.530	0.223	0.131	0.130	0.194	0.183	0.106	0.010	0.086	0.454
ROC5	0.541	0.421	0.057	0.160	−0.037	0.103	0.152	0.192	0.041	0.572
Eigenvalue (before rotation)	14.075	1.857	1.544	1.370	1.307	1.184	1.100	1.087	1.048	–
Variance explained (%) (before rotation)	31.278%	4.127%	3.430%	3.044%	2.905%	2.632%	2.444%	2.416%	2.329%	–
Cumulative variance explained (%) (before rotation)	31.278%	35.404%	38.835%	41.879%	44.784%	47.415%	49.860%	52.276%	54.605%	–
Eigenvalue (after rotation)	6.709	4.628	2.184	2.047	1.912	1.822	1.791	1.758	1.708	–
Variance explained (%) (after rotation)	14.909%	10.284%	4.853%	4.550%	4.248%	4.049%	3.979%	3.906%	3.796%	–
Cumulative variance explained (%) (after rotation)	14.909%	25.193%	30.046%	34.595%	38.844%	42.892%	46.872%	50.777%	54.574%	–
KMO value	0.947									–
Bartlett’s Sphericity test value	8836.550									–
Degrees of freedom	990									–
*p*-value	0.000									–

### 3.6 Confirmatory factor analysis

Based on the analysis of factor loadings, it can be seen that the relationships between the measurement items and latent variables are generally significant. First, the standardized loading coefficients reflect the degree of association between each measurement item and its corresponding latent variable. Most measurement items have high standardized loading coefficients, indicating a strong positive correlation with their latent variables. For example, the standardized loading coefficient for TSPF2 (Teacher’s Sense of Professional Flourishing) with its latent variable is 0.683, demonstrating the strong explanatory power of this measurement item in reflecting teacher professional flourishing. Additionally, the z-value (CR value) is a statistical indicator used to assess whether the factor loadings are significant. All the z-values of the measurement items are significantly greater than 1.96, suggesting that their factor loadings have reached statistical significance. For instance, the z-value for TSPF5 is 8.720, and the z-value for OES5 is 18.446, both far exceeding 1.96, further confirming the important role these measurement items play in the constructs. Therefore, it can be concluded that all the measurement items in the table effectively measure the corresponding latent variables, and these latent variables demonstrate strong reliability and validity in the theoretical model (Table 5).

**Table 5:** Confirmatory factor analysis.

Factor	Item	Unstandardized loading coefficient	Std. error	*z* (CR)	*p*	Std. estimate	SMC	AVE	CR
TSPF	TSPF2	1.000	–	–	–	0.683	0.467	0.434	0.605
	TSPF5	0.979	0.112	8.720	0.000	0.633	0.401		
GAI	GAI1	1.000	–	–	–	0.570	0.325	0.430	0.598
	GAI4	1.533	0.216	7.094	0.000	0.731	0.535		
OES	OES1	1.000	–	–	–	0.774	0.598	0.554	0.860
	OES2	0.978	0.059	16.604	0.000	0.726	0.528		
	OES3	1.166	0.065	17.919	0.000	0.776	0.602		
	OES4	0.800	0.056	14.316	0.000	0.637	0.406		
	OES5	1.131	0.061	18.446	0.000	0.796	0.633		
OTS	OTS4	1.000	–	–	–	0.718	0.515	0.470	0.639
	OTS5	0.771	0.057	13.571	0.000	0.652	0.426		
RA	RA2	1.000	–	–	–	0.731	0.534	0.504	0.752
	RA3	0.821	0.061	13.501	0.000	0.649	0.421		
	RA5	0.964	0.062	15.478	0.000	0.747	0.557		
TA	TA1	1.000	–	–	–	0.653	0.427	0.479	0.647
	TA4	1.228	0.095	12.955	0.000	0.728	0.531		
BTR	BTR1	1.000	–	–	–	0.706	0.498	0.530	0.693
	BTR4	1.205	0.096	12.526	0.000	0.750	0.562		
TE	TE3	1.000	–	–	–	0.570	0.325	0.315	0.479
	TE5	0.951	0.113	8.455	0.000	0.552	0.305		
ROC	ROC3	1.000	–	–	–	0.679	0.461	0.529	0.691
	ROC5	1.106	0.075	14.705	0.000	0.773	0.597		

Finally, the Squared Multiple Correlation (SMC) values represent the shared variance between each measurement item and all other items. The data shows that most measurement items have high SMC values, particularly OES5 and RA5, with SMC values of 0.633 and 0.557, respectively. This indicates that these measurement items have significant shared variance with their latent variables, effectively reflecting the changes in the underlying constructs.

The CFA model in this study shows good fit. The χ^2^ test results are χ^2^ = 331.077. The *χ*^2^/df ratio is 1.914, indicating a good fit (threshold < 3). The Goodness of Fit Index (GFI) is 0.945, RMSEA is 0.043, CFI is 0.963, NFI is 0.926, and NNFI is 0.950, all exceeding 0.9, indicating strong model fit. Additional fit indices show TLI = 0.950, AGFI = 0.919, IFI = 0.963, PGFI = 0.646, PNFI = 0.694, and PCFI = 0.721, all above 0.5. SRMR = 0.031, confirming excellent model fit (Table 5).

## 4 Results

The discriminant validity analysis (Table 6) shows that the square root of AVE for TSPF is 0.659, which is greater than the highest correlation coefficient of 0.443, indicating good discriminant validity. For GAI, the square root of AVE is 0.656, higher than the highest correlation of 0.340, confirming good discriminant validity. The square root of AVE for OES is 0.744, exceeding the highest correlation of 0.665, indicating good discriminant validity.

**Table 6:** Discriminant validity analysis.

	TSPF	GAI	OES	OTS	RA	TA	BTR	TE	ROC
TSPF	0.659								
GAI	0.265	0.656							
OES	0.419	0.319	0.744						
OTS	0.329	0.311	0.665	0.686					
RA	0.443	0.325	0.653	0.591	0.710				
TA	0.386	0.340	0.602	0.577	0.594	0.692			
BTR	0.283	0.279	0.553	0.486	0.500	0.459	0.728		
TE	0.302	0.314	0.425	0.347	0.419	0.424	0.408	0.561	
ROC	0.309	0.315	0.643	0.596	0.658	0.484	0.511	0.429	0.727

The blue numbers on the diagonal represent the square root of AVE.

For OTS, the square root of AVE is 0.686, which is higher than the highest correlation of 0.665, demonstrating good discriminant validity. RA’s square root of AVE is 0.710, greater than the highest correlation of 0.658, showing good discriminant validity. TA’s square root of AVE is 0.692, higher than the highest correlation of 0.602, confirming good discriminant validity. For BTR, the square root of AVE is 0.728, exceeding the highest correlation of 0.553, indicating good discriminant validity. The square root of AVE for TE is 0.561, which is greater than the highest correlation of 0.429, suggesting good discriminant validity. Finally, the square root of AVE for ROC is 0.727, which is higher than the highest correlation of 0.658, indicating good discriminant validity.

This study analyzes the impact of six independent variables and two mediating variables on the dependent variable, Teacher’s Sense of Professional Flourishing (TSPF). The variables are Research Atmosphere (RA), Teaching Atmosphere (TA), Generative Artificial Intelligence Literacy (GAI), Organizational Emotional Support (OES), Teaching Efficiency (TE), Research and Teaching Balance (BTR), and Organizational Tool Support (OTS). The data analysis results indicate that GAI, RA, TA, OES, and TE significantly contribute to enhancing TSPF, while BTR and OTS do not have a significant impact on TSPF. Control variables such as gender, age, income, and the frequency of using generative artificial intelligence tools do not significantly affect TSPF (Table 7).

**Table 7:** Mediation analysis.

Item	Symbol	Meaning	Effect	95% CI		SE	*z*/*t*	*p*	Conclusion
				Lower	Upper				
BTR → ROC → TSPF	a*b	Indirect Effect	−0.018	−0.086	0.054	0.036	−0.500	0.617	The Mediation Effect is Not Significant
BTR → ROC	a	X → M	0.316	0.235	0.397	0.041	7.659	0.000	
ROC → TSPF	b	M → Y	−0.056	−0.143	0.031	0.044	−1.267	0.206	
BTR → TSPF	c’	Direct Effect	−0.063	−0.147	0.022	0.043	−1.462	0.144	
BTR → TSPF	c	Total Effect	−0.051	−0.131	0.029	0.041	−1.253	0.211	
BTR → TE → TSPF	a*b	Indirect Effect	0.029	0.008	0.083	0.019	1.522	0.128	Total Mediation
BTR → TE	a	X → M	0.159	0.081	0.237	0.040	4.001	0.000	
TE → TSPF	b	M → Y	0.185	0.094	0.275	0.046	4.010	0.000	
BTR → TSPF	c’	Direct Effect	−0.063	−0.147	0.022	0.043	−1.462	0.144	
BTR → TSPF	c	Total Effect	−0.051	−0.131	0.029	0.041	−1.253	0.211	
TA → ROC → TSPF	a*b	Indirect Effect	−0.000	−0.013	0.029	0.010	−0.016	0.988	The Mediation Effect is Not Significant
TA → ROC	a	X → M	0.003	−0.106	0.112	0.055	0.049	0.961	
ROC → TSPF	b	M → Y	−0.056	−0.143	0.031	0.044	−1.267	0.206	
TA → TSPF	c’	Direct Effect	0.169	0.063	0.275	0.054	3.121	0.002	
TA → TSPF	c	Total Effect	0.194	0.087	0.302	0.055	3.562	0.000	
TA → TE → TSPF	a*b	Indirect Effect	0.026	0.000	0.084	0.022	1.168	0.243	Partial Mediation
TA → TE	a	X → M	0.138	0.034	0.243	0.053	2.598	0.010	
TE → TSPF	b	M → Y	0.185	0.094	0.275	0.046	4.010	0.000	
TA → TSPF	c’	Direct Effect	0.169	0.063	0.275	0.054	3.121	0.002	
TA → TSPF	c	Total Effect	0.194	0.087	0.302	0.055	3.562	0.000	
RA → ROC → TSPF	a*b	Indirect Effect	−0.024	−0.116	0.079	0.050	−0.482	0.630	The Mediation Effect is Not Significant
RA → ROC	a	X → M	0.430	0.340	0.520	0.046	9.396	0.000	
ROC → TSPF	b	M → Y	−0.056	−0.143	0.031	0.044	−1.267	0.206	
RA → TSPF	c’	Direct Effect	0.159	0.064	0.254	0.048	3.279	0.001	
RA → TSPF	c	Total Effect	0.132	0.044	0.221	0.045	2.928	0.004	
RA → TE → TSPF	a*b	Indirect Effect	−0.002	−0.032	0.024	0.013	−0.184	0.854	The Mediation Effect is Not Significant
RA → TE	a	X → M	−0.013	−0.100	0.073	0.044	−0.301	0.764	
TE → TSPF	b	M → Y	0.185	0.094	0.275	0.046	4.010	0.000	
RA → TSPF	c’	Direct Effect	0.159	0.064	0.254	0.048	3.279	0.001	
RA → TSPF	c	Total Effect	0.132	0.044	0.221	0.045	2.928	0.004	
OTS → ROC → TSPF	a*b	Indirect Effect	−0.002	−0.022	0.023	0.010	−0.238	0.812	The Mediation Effect is Not Significant
OTS → ROC	a	X → M	0.043	−0.058	0.145	0.052	0.842	0.400	
ROC → TSPF	b	M → Y	−0.056	−0.143	0.031	0.044	−1.267	0.206	
OTS → TSPF	c’	Direct Effect	0.097	−0.002	0.196	0.050	1.932	0.054	
OTS → TSPF	c	Total Effect	0.115	0.015	0.215	0.051	2.258	0.024	
OTS → TE → TSPF	a*b	Indirect Effect	0.020	−0.001	0.068	0.018	1.109	0.268	Total Mediation
OTS → TE	a	X → M	0.108	0.011	0.206	0.050	2.178	0.030	
TE → TSPF	b	M → Y	0.185	0.094	0.275	0.046	4.010	0.000	
OTS → TSPF	c’	Direct Effect	0.097	−0.002	0.196	0.050	1.932	0.054	
OTS → TSPF	c	Total Effect	0.115	0.015	0.215	0.051	2.258	0.024	
OES → ROC → TSPF	a*b	Indirect Effect	−0.007	−0.052	0.026	0.020	−0.365	0.715	The Mediation Effect is Not Significant
OES → ROC	a	X → M	0.127	0.059	0.195	0.035	3.665	0.000	
ROC → TSPF	b	M → Y	−0.056	−0.143	0.031	0.044	−1.267	0.206	
OES → TSPF	c’	Direct Effect	0.071	0.004	0.138	0.034	2.078	0.038	
OES → TSPF	c	Total Effect	0.070	0.003	0.137	0.034	2.051	0.041	
OES → TE → TSPF	a*b	Indirect Effect	0.006	−0.010	0.042	0.013	0.479	0.632	The Mediation Effect is Not Significant
OES → TE	a	X → M	0.034	−0.031	0.100	0.033	1.023	0.307	
TE → TSPF	b	M → Y	0.185	0.094	0.275	0.046	4.010	0.000	
OES → TSPF	c’	Direct Effect	0.071	0.004	0.138	0.034	2.078	0.038	
OES → TSPF	c	Total Effect	0.070	0.003	0.137	0.034	2.051	0.041	
GAI → ROC → TSPF	a*b	Indirect Effect	−0.006	−0.039	0.012	0.012	−0.479	0.632	The Mediation Effect is Not Significant
GAI → ROC	a	X → M	0.105	0.018	0.192	0.044	2.380	0.018	
ROC → TSPF	b	M → Y	−0.056	−0.143	0.031	0.044	−1.267	0.206	
GAI → TSPF	c’	Direct Effect	0.144	0.058	0.231	0.044	3.287	0.001	
GAI → TSPF	c	Total Effect	0.170	0.085	0.256	0.044	3.903	0.000	
GAI → TE → TSPF	a*b	Indirect Effect	0.032	0.006	0.071	0.017	1.848	0.065	Partial Mediation
GAI → TE	a	X → M	0.173	0.090	0.257	0.043	4.072	0.000	
TE → TSPF	b	M → Y	0.185	0.094	0.275	0.046	4.010	0.000	
GAI → TSPF	c’	Direct Effect	0.144	0.058	0.231	0.044	3.287	0.001	
GAI → TSPF	c	Total Effect	0.170	0.085	0.256	0.044	3.903	0.000	

First, RA, TA, GAI, OES, and TE show significant effects on TSPF. Specifically, RA has a direct effect of 0.159 with a *p*-value of 0.001, indicating that the research atmosphere significantly enhances TSPF.

GAI has a direct positive effect on TSPF, with a *p* < 0.05, indicating that generative AI literacy (GAI) significantly influences teachers’ professional flourishing (TSPF) in a positive direction. *Hypothesis 1 is supported.* The higher the level of generative AI literacy, the stronger the sense of professional flourishing among teachers. Teachers’ AI literacy significantly enhances their satisfaction with autonomy, competence, and relatedness, which are closely linked to job satisfaction and professional wellbeing (Huang and Zhao, 2025). This, in turn, indirectly promotes improvements in career-related outcomes. Additionally, AI literacy is an essential component of teachers’ professional knowledge, as it enhances their decision-making abilities and technical application skills in teaching practice. Teachers with higher AI literacy are better able to effectively integrate AI tools, improving the quality and effectiveness of classroom teaching (Sperling et al., 2024), thereby enhancing their professional flourishing.

OES has a direct positive effect on TSPF, with a *p* < 0.05, indicating that organizational emotional support (OES) significantly influences teachers’ professional flourishing (TSPF) in a positive direction. Therefore, *Hypothesis 2 is supported*: The higher the level of emotional support provided by the organization, the stronger the teachers’ sense of professional flourishing. Organizational emotional support significantly enhances teachers’ satisfaction with autonomy, competence, and relatedness (Hakanen et al., 2006), which are closely linked to job satisfaction and professional wellbeing, thereby indirectly promoting improvements in career-related outcomes. Additionally, emotional support plays a critical role in reducing work-related stress and improving emotional health. Teachers receiving emotional support from the organization are more likely to experience higher job satisfaction, stronger professional commitment, and enhanced mental wellbeing, ultimately leading to a higher sense of professional flourishing.

Hypothesis 3, which posited that organizational tool support (OTS) has a direct positive effect on teachers’ professional flourishing (TSPF), is not supported by the data. The *p*-value for the relationship between OTS and TSPF was found to be greater than 0.05, indicating that the effect is not statistically significant. One possible explanation for the non-significant result could be that while organizational tool support, such as teaching materials, technological platforms, and research resources, is important, its influence on teachers’ professional flourishing might be more indirect or conditional on other factors. For example, teachers may require not only the tools but also the appropriate training and institutional culture to use them effectively. If the organizational tool support is not coupled with adequate professional development or integration into the teaching and research environment, it may fail to have a significant impact on teachers’ sense of professional flourishing. Another potential reason for the lack of significance could be that the relationship between tool support and professional flourishing is moderated by other factors such as the level of AI literacy, teaching experience, or personal attitudes toward technology. In cases where teachers feel adequately supported in other areas, such as emotional support or autonomy, the additional impact of tool support on their professional flourishing may be diminished.

Hypothesis 4, which suggests that the research atmosphere (RA) has a direct positive effect on teachers’ professional flourishing (TSPF), was not supported by the data. According to the data analysis, the relationship between the research atmosphere and professional flourishing did not reach statistical significance (*p*-value greater than 0.05). One possible reason for this result is that teachers’ professional flourishing is not solely dependent on the research atmosphere; it is also influenced by other factors in the work environment. Teachers may rely more on teaching activities rather than research activities, especially in more practice-oriented disciplines, where the impact of the research atmosphere on professional flourishing is relatively small. In academic disciplines that are research-oriented, teachers may place more emphasis on teaching support or the work environment rather than solely on the research atmosphere. Furthermore, the relationship between teachers’ professional flourishing and the research atmosphere may be the result of long-term accumulation, rather than an immediate effect. Even when the research atmosphere is positive, teachers may need more time to feel its impact on their professional flourishing. The effect of the research atmosphere may become more significant after a certain period, especially in terms of research achievement accumulation and career development. Therefore, the lack of a significant relationship in the short term may also be due to the fact that the effect of the research atmosphere has not yet fully manifested.

Hypothesis 5, which posits that the teaching atmosphere (TA) has a direct positive effect on teachers’ professional flourishing (TSPF), is supported by the data. The direct effect of TA on TSPF is 0.169, with a *p*-value of 0.002, indicating that the teaching atmosphere significantly influences teachers’ professional flourishing. This finding suggests that a positive and supportive teaching environment enhances teachers’ job satisfaction, sense of achievement, and overall professional wellbeing. Teachers working in a positive teaching atmosphere tend to experience greater professional engagement, increased motivation, and a stronger sense of accomplishment, which directly contribute to their professional flourishing.

*Hypothesis 6 is not supported by the data*: the analysis revealed no significant relationship between the ability to balance teaching and research and teachers’ professional flourishing. One possible reason for this is that the ability to balance teaching and research is not the sole factor determining teachers’ professional flourishing. Teachers’ professional flourishing may be more influenced by other factors, such as the work environment, social support, or personal expectations, which may have a more direct and significant impact. Furthermore, the effect of work-life balance might become apparent only after a longer period, and its immediate impact on professional flourishing may not be as noticeable. Therefore, the influence of work-life balance on professional flourishing may require more time to become evident.

*Hypothesis 7 is supported*: The analysis shows that teaching efficiency (TE) has a direct positive effect on teachers’ professional flourishing (TSPF) with a coefficient of 0.169 and a p-value of 0.002, which indicates a statistically significant relationship. This suggests that the higher the teaching efficiency, the stronger the teachers’ sense of professional flourishing. Efficient teaching allows teachers to achieve their teaching goals more effectively, reducing stress and enhancing job satisfaction, which in turn positively impacts their professional wellbeing and overall professional flourishing.

*Hypothesis 8 is not supported*: The data analysis shows that there is no significant positive relationship between research output capacity and teachers’ sense of professional flourishing. One possible reason is that teachers’ sense of professional flourishing is not solely dependent on the quantity or quality of research output, but is more influenced by a sense of achievement and student feedback in teaching work, especially in positions with heavy teaching tasks. Teachers may feel that while research output can enhance academic reputation, its direct impact on professional flourishing is relatively small, particularly in the short term. The accumulation of research results does not immediately contribute to enhancing teachers’ psychological satisfaction or sense of achievement from work. Therefore, the lack of a significant relationship in the short term may be because the actual impact of research output has not yet been fully realized.

Additionally, this study identifies four mediating pathways.

First, Research and Teaching Balance (BTR) influences Teacher’s Sense of Professional Flourishing (TSPF) through Teaching Efficiency (TE). Finding a good balance between teaching and research helps teachers allocate their time and energy more efficiently, thereby improving teaching effectiveness. When teachers are able to manage their time effectively, they can complete more teaching tasks within limited time, improving the quality of classroom instruction. This efficient teaching performance not only enhances students’ learning outcomes but also strengthens teachers’ sense of accomplishment in teaching. Furthermore, high teaching efficiency boosts teachers’ self-efficacy (Bisht et al., 2026). When teachers see the results of their work and are able to help students learn better, their professional identity and confidence naturally grow. These positive emotions and a sense of identity directly affect their job satisfaction and mental health, creating a positive feedback loop. In other words, the balance between teaching and research (Xiao and Zheng, 2025), by enhancing teaching efficiency, improves teachers’ work performance, reduces stress caused by excessive focus or conflicts between teaching and research, and thus indirectly enhances their sense of professional flourishing.

Second, Teaching Atmosphere (TA) influences TSPF through TE. First, the teaching atmosphere has a direct impact on teachers’ work attitudes and performance. A supportive and positive teaching atmosphere can motivate teachers to invest more energy and creativity in their teaching, thereby improving their teaching efficiency. When teachers feel supported by the school and colleagues, they are able to organize teaching activities more effectively in the classroom, optimize teaching methods, and enhance student engagement and classroom interaction, all of which directly improve teaching efficiency (Qu, 2024; Eryilmaz et al., 2025). Secondly, improving teaching efficiency directly enhances teachers’ sense of professional achievement and job satisfaction. Efficient teaching helps teachers better achieve their teaching goals, gaining a greater sense of accomplishment, which in turn increases their work engagement and professional identity. These positive emotions and sense of recognition contribute to the enhancement of teachers’ professional flourishing. Therefore, the teaching atmosphere indirectly promotes teachers’ professional flourishing by improving teaching efficiency.

Third, Organizational Tool Support (OTS) influences Teacher’s Sense of Professional Flourishing (TSPF) through Teaching Efficiency (TE). First, organizational tool support is primarily reflected in the teaching resources, technological equipment, and platforms provided by schools. These supports directly improve the teaching conditions for teachers. When teachers receive sufficient teaching resources and tools, they are able to prepare teaching materials more smoothly, optimize classroom designs, and integrate modern educational technology into their teaching, thus enhancing teaching efficiency and classroom interaction quality. Empirical research has shown that organizational support provided by schools is significantly positively correlated with teaching efficiency, indicating that institutional support helps teachers efficiently carry out teaching activities (Delgra, 2026). Second, improving teaching efficiency enhances teachers’ sense of achievement and job satisfaction in teaching tasks, which are important components of professional flourishing. Studies show that when teachers are provided with the necessary materials and equipment support, their satisfaction with the work environment significantly increases. Higher job satisfaction is closely related to stronger professional wellbeing and professional identity. Therefore, organizational tool support, by improving teaching efficiency, creates a smoother teaching experience and a greater sense of achievement for teachers, indirectly promoting their professional flourishing.

Fourth, Generative Artificial Intelligence Literacy (GAI) has a direct influence on teachers’ sense of professional flourishing (TSPF) through teaching efficiency (TE). First, GAI equips teachers with the necessary skills and knowledge to integrate advanced technological tools into their teaching practices. Teachers with higher GAI are more adept at utilizing AI tools to optimize their teaching methods, making them more effective and efficient in the classroom. Research by Sperling et al. (2024) emphasizes that teachers with higher AI literacy can streamline lesson planning, enhance student engagement, and optimize classroom interaction, all of which contribute to improved teaching outcomes and greater teaching efficiency. Second, GAI enhances teachers’ ability to manage classroom activities and educational tasks in a more dynamic and personalized manner. By leveraging AI tools, teachers can customize learning experiences, provide real-time feedback, and foster an interactive classroom environment. This capability not only boosts teaching efficiency but also allows teachers to achieve better educational outcomes with fewer resources, reducing time spent on repetitive tasks. The resulting increase in teaching efficiency leads to a greater sense of professional achievement, job satisfaction, and self-efficacy, all of which are key components of TSPF. Furthermore, as teachers’ AI literacy improves, they become more confident in their ability to adapt to the evolving technological landscape, which reinforces their sense of professional identity and career satisfaction (Huang and Zhao, 2025). This alignment with modern educational practices supports their career development, reduces stress, and fosters a sense of belonging within the academic community. Thus, by enhancing teaching efficiency, GAI plays a critical role in the overall professional flourishing of teachers, aligning with the findings that link technology adoption to improved wellbeing and job satisfaction.

## 5 Discussion

### 5.1 Theoretical insights

This study provides theoretical insights from a psychological perspective, revealing the multidimensional composition of Teacher’s Sense of Professional Flourishing (TSPF) and the complex psychological mechanisms behind it.

First, teacher professional flourishing (TSPF) is influenced by both internal and external factors. External support factors, such as the research atmosphere (RA), teaching atmosphere (TA), and organizational emotional support (OES), along with personal ability factors, such as teaching efficiency (TE) and generative artificial intelligence literacy (GAI), have direct effects on TSPF. These findings indicate that teachers’ professional flourishing is not only determined by their intrinsic motivation but also by the external environment, particularly emotional support and resource support. A positive research atmosphere and teaching atmosphere, as well as emotional support from the organization, can significantly enhance teachers’ sense of belonging and self-efficacy, thereby improving their job satisfaction and sense of achievement. In such a supportive environment, teachers are more likely to receive psychological support and feedback, which contributes to their overall professional flourishing.

Second, external support systems play a crucial role in the study of teachers’ professional flourishing. External factors such as research and teaching balance (BTR), teaching atmosphere (TA), and organizational tool support (OTS) indirectly promote professional flourishing by enhancing teachers’ work efficiency, further validating social support theory. These external support factors provide necessary resources and emotional support, helping teachers alleviate work-related stress and boosting their work motivation, thereby improving their job satisfaction and professional identity. In such a supportive environment, teachers experience a sense of belonging and self-worth, which not only enhances their professional confidence but also contributes to their overall professional wellbeing.

Third, Generative Artificial Intelligence Literacy (GAI) is one of the factors that enhance teachers’ professional flourishing, reflecting the close relationship between technological literacy and psychological wellbeing. Teachers’ GAI, particularly in the use of generative AI, has become not only a tool for improving teaching efficiency but also has a profound impact on their mental health and job satisfaction. As the educational environment becomes increasingly intelligent, teachers’ mastery of modern technologies not only enhances teaching effectiveness but also increases their sense of control and confidence in the teaching process. This has a positive effect on reducing the anxiety caused by work pressure. GAI helps teachers efficiently reduce the time spent on extensive lesson preparation, allowing them to focus on teaching quality and student interaction. This reduces the time and energy pressure, thereby lowering the risk of job burnout. This also indicates that the future professional development and psychological wellbeing of teachers will depend not only on traditional teaching skills but also on high levels of technological adaptability, especially in responding to the rapidly evolving educational technologies. Moreover, teachers who master generative AI are typically more flexible in adapting to changes in the teaching environment and can use technology to innovate in their teaching. This innovation not only enhances students’ learning experiences but also provides teachers with more professional achievement. Through AI technologies, teachers can provide personalized learning experiences for students, improving their academic performance and thus gaining greater satisfaction and recognition in their teaching. These factors are closely related to professional flourishing, further strengthening teachers’ sense of belonging to the education profession and their long-term commitment.

Fourth, although the direct effects of BTR (Teaching-Research Balance) and OTS (Organizational Tool Support) on teachers’ professional flourishing (TSPF) are not significant, they indirectly influence TSPF through enhancing teaching efficiency (TE). This suggests that teaching-research balance and tool support play a role in improving teachers’ work performance, but their impact on teachers’ job satisfaction and sense of achievement is not realized by directly altering teachers’ professional identity or psychological state. Instead, it is achieved indirectly through improvements in their teaching efficiency and work outcomes. Therefore, the impact pathways of BTR and OTS on teachers’ professional flourishing may be more complex and require the accumulation of a longer period or the coordination of external contextual factors to reveal their positive effects.

### 5.2 Practical insights

This study provides the following practical insights:

First, the significant impact of the research atmosphere on teachers’ professional flourishing suggests that educational administrators should focus on optimizing the academic research environment. Improving the research atmosphere not only helps increase teachers’ interest in research and their sense of involvement but also enhances their academic achievement by providing more resources and support. Therefore, schools should strengthen the development of research platforms, encourage interdisciplinary cooperation, optimize resource allocation, and foster an environment that encourages innovation and the transformation of research outcomes. A positive research environment not only enhances teachers’ academic research abilities but also helps them find a better balance between teaching and research, thereby improving their professional flourishing.

Second, the teaching atmosphere also plays a crucial role in enhancing teachers’ professional flourishing. A positive teaching atmosphere provides a supportive work environment that can ignite teachers’ enthusiasm and motivation for innovation. Studies show that when teachers work in a cooperative, respectful, and supportive environment, they are able to complete teaching tasks more efficiently and experience greater professional achievement. Therefore, educational administrators should promote collaboration and interaction among teachers, encourage team spirit, and strengthen communication and knowledge sharing.

Additionally, the impact of Generative Artificial Intelligence Literacy (GAI) on teachers’ professional flourishing offers new insights for educational administrators. At the individual level, educational administrators should recognize the role of generative artificial intelligence (GAI) literacy in enhancing teachers’ professional flourishing. Through policies, resource allocation, and professional development programs, administrators should promote teachers’ learning and growth in this area. Teachers should receive ongoing training in generative AI technology to improve their ability to use AI tools effectively in teaching. This will not only enhance teaching effectiveness but also increase teachers’ sense of professional achievement and job satisfaction. At the organizational level, educational administrators should create a supportive work environment by providing the necessary technical resources and training opportunities to help teachers enhance their generative AI literacy. Furthermore, schools should encourage collaboration and experience sharing among teachers, promoting interdisciplinary cooperation, strengthening teamwork, and fostering knowledge sharing. This approach will help teachers master generative AI tools, increase their work support and sense of belonging, and further promote their professional flourishing. At the policy level, educational institutions should establish long-term development strategies that ensure the enhancement of generative AI literacy is supported at all stages of teachers’ professional development. From policy guidance to concrete investments in funds and resources, educational administrators need to take comprehensive measures to provide better technological support and development opportunities for teachers, maximizing their professional satisfaction and wellbeing.

At the same time, the positive impact of teaching efficiency (TE) on teachers’ professional flourishing indicates that improving work efficiency is a crucial way to enhance their professional flourishing. To begin with, at the policy level, educational administrators should define clear goals and measures for improving teaching efficiency, establish specialized teams to regularly evaluate teaching processes, and ensure a reasonable balance between teaching content and teachers’ workloads to avoid overwork. Second, at the resource level, schools should provide modern teaching equipment and technological platforms to ensure that teachers have access to efficient teaching tools. By properly allocating teaching resources, teachers can reduce preparation time and spend more time engaging with students, thereby increasing teaching efficiency and student participation. Third, at the training and professional development level, schools should regularly organize training on modern educational technologies and teaching methods. Specifically, they should focus on enhancing teachers’ generative AI literacy, helping them master efficient teaching tools to improve teaching interaction quality and work efficiency. Besides, at the school culture and support system level, schools should create a supportive and collaborative environment, encouraging teachers to share experiences and engage in interdisciplinary cooperation. School leaders should regularly communicate with teachers, understand their needs, and provide support to ensure that teachers maintain a good working state.

Finally, the impact of organizational emotional support on teachers’ professional flourishing offers valuable insights for educational administrators. Administrators should prioritize teachers’ mental health and emotional needs, recognizing their crucial role in professional flourishing. Schools should provide emotional support systems such as counseling and staff care programs to reduce burnout and enhance job satisfaction, which in turn fosters professional growth. Furthermore, administrators should promote collaboration and leadership care. Encouraging teamwork and cross-disciplinary cooperation strengthens emotional bonds and work support. Regular communication from school leaders can also improve teachers’ sense of belonging and self-efficacy, further boosting professional flourishing. Moreover, emotional support should be integrated with professional development. Offering training opportunities can help teachers improve their teaching and research skills, reinforcing their professional identity and accomplishment. Besides, emotional support’s benefits may not be immediate, but long-term investment leads to sustained improvements in job satisfaction and wellbeing. Administrators should develop strategies to provide continuous support, ensuring teachers can thrive in a stable, nurturing environment.

## 6 Conclusion

This study is based on the self-determination theory and social support theory, constructing a theoretical framework for teacher professional flourishing. Data were collected through a questionnaire survey to innovatively explore the impact of personal ability factors, such as generative artificial intelligence literacy, and external factors on teachers’ sense of professional flourishing. The results reveal that teachers’ sense of professional flourishing is influenced by both internal factors and external support factors. Specifically, external support factors, such as the research atmosphere, teaching atmosphere, and organizational emotional support, along with personal ability factors, such as teaching efficiency and generative artificial intelligence literacy, all have direct effects on professional flourishing. Furthermore, the study identifies four mediation pathways, showing how these factors indirectly affect teachers’ professional flourishing through teaching efficiency as a mediating variable. Specifically, generative artificial intelligence literacy significantly improves teachers’ professional flourishing by enhancing teaching efficiency, while the research atmosphere, teaching atmosphere, and organizational emotional support also significantly improve teachers’ professional flourishing through the enhancement of teaching efficiency. These findings suggest that teachers’ sense of professional flourishing is not only directly influenced by personal ability factors but also positively impacted by external support systems, particularly through the enhancement of teaching efficiency.

Although this study reveals the significant impact of generative artificial intelligence literacy and other related factors on teachers’ professional flourishing, several limitations affect the generalizability and depth of the findings. First, the data in this study were primarily collected from a specific region, which may limit the applicability of the results across different geographical and cultural contexts. Teachers in different regions, cultures, and educational systems may face distinct career development challenges, and the mechanisms behind their professional flourishing may differ. Therefore, future research could consider cross-regional and cross-cultural samples to further validate the generalizability of the conclusions. Additionally, this study relied solely on survey data, which may be subject to self-report bias. Social desirability bias during the survey completion process could influence the accuracy of the results. Future research could combine qualitative methods, such as in-depth interviews or focus group discussions, to obtain richer data and a deeper understanding of the factors affecting teachers’ professional flourishing.

## Data Availability

The raw data supporting the conclusions of this article will be made available by the authors, without undue reservation.
